# Bis[(2-bromo­phen­yl)di­cyclo­hexyl­phosphane-κ^2^*Br*,*P*]di­chlorido­ruthenium(II) methanol 1.6-solvate

**DOI:** 10.1107/S2414314626010862

**Published:** 2026-10-23

**Authors:** Monsuru T. Kelani, Eric C. Hosten, Alfred Muller, Koop Lammertsma

**Affiliations:** a Department of Chemical Sciences, University of Johannesburg, Auckland Park, Johannesburg 2006, South Africa; b Department of Chemistry, Nelson Mandela University, Summerstrand, Port Elizabeth 6001, South Africa; Benemérita Universidad Autónoma de Puebla, México

**Keywords:** crystal structure, ruthenium, monoclinic system, octa­hedral coordination

## Abstract

The title compound, [RuCl_2_(C_18_H_26_BrP)_2_]·1.6CH_3_OH, crystallizes in the monoclinic space group *P*2*/n* with two and a half mol­ecules plus four mol­ecules of methanol solvent in the asymmetric unit (*Z*′ = 5/2, *Z* = 10). Distortion of the octahedral coordination environment results in the slight variation of bond lengths and bond angles from ideal values. The crystal packing is reinforced by classical hydrogen bonding, O—H⋯*X* (where *X* = O or Cl) and non-classical hydrogen bonding inter­actions such as C—H⋯Cl.

## Abstract 2

The title Ru^II^ complex crystallizes with two and a half mol­ecules (*Z*′ = 5/2) plus four mol­ecules of methanol solvent in the asymmetric unit.

## Graphical abstract

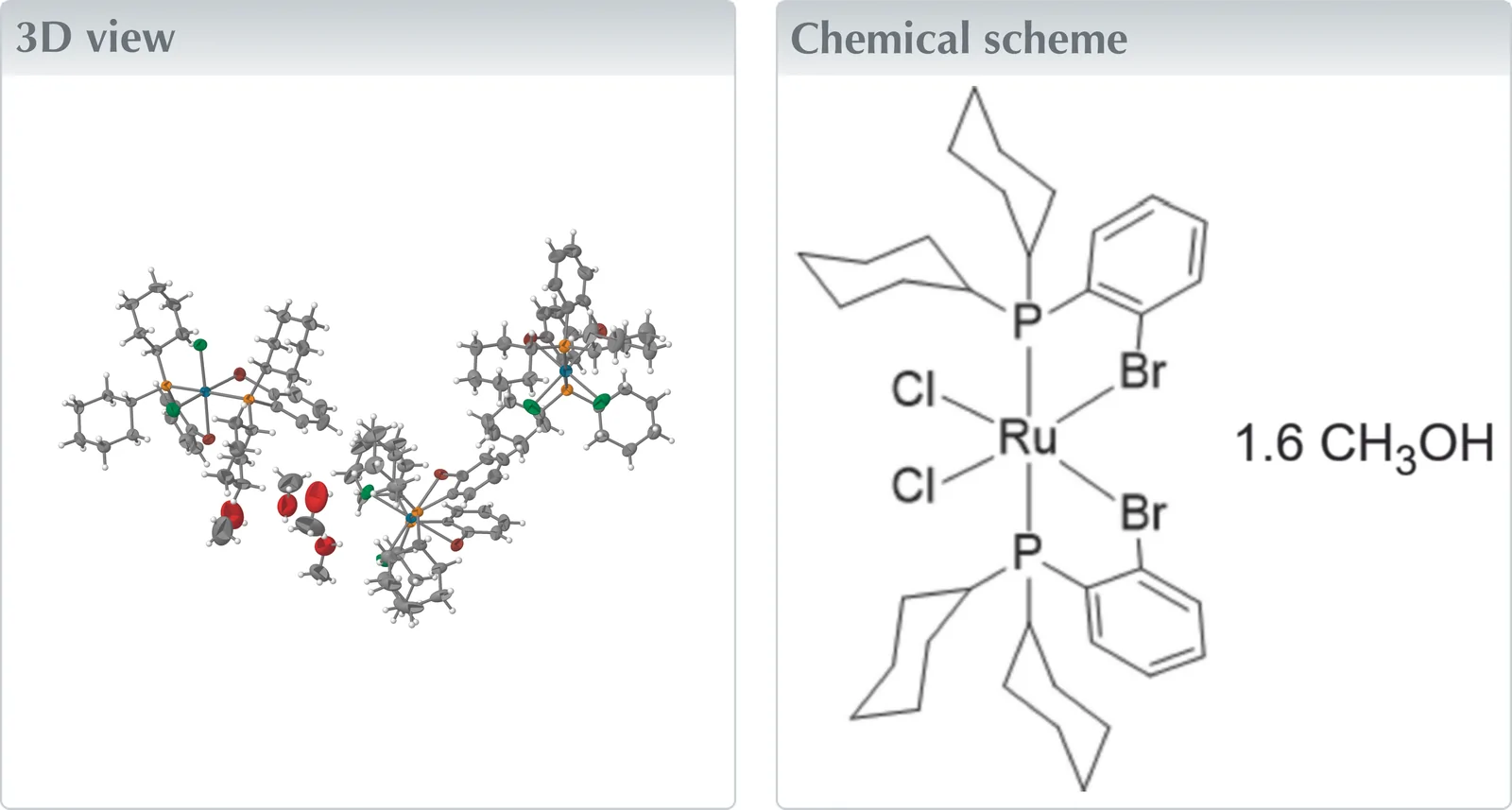

## Structure description

Phospho­rus-bound ruthenium complexes display widespread applications in organometallic chemistry and homogeneous catalysis to influence reactivity and selectivity in most cases (Balakrishna *et al.*, 2000 ▸). They are often exploited in reductive elimination and oxidative addition for making and breaking C—H bonds (Ittel *et al.*, 1978 ▸; Hartwig *et al.*, 1991*a* ▸) as well as the cleavage of N—H and O—H bonds (Hartwig *et al.*, 1991*b* ▸). Hence, these and many more potentials have attracted a growing inter­est in exploring phospho­rus-bound ruthenium complexes.

In this report, the title compound forms an octa­hedral complex defined by two mol­ecules of (2-bromo­phen­yl)di­cyclo­hexyl­phosphane ligands and two chlorido ligands. In order to avoid steric hindrance, the phosphane ligands are positioned *trans* to each other while the bromo substituents of the phenyl rings of the phosphanes also form coordination with the ruthenium atom, hence, the phosphanes coordinate as bidentate ligands. As a result, the bromo groups coordinate in a *cis* arrangement, like the chlorido ligands (Fig. 1 ▸). This is achieved through twisting of the bromo­phenyl rings, resulting in a distorted octa­hedral geometry coordination environment. The distortion leads to a slight variation in bond lengths between the Ru^II^ atom and the ligands from ideal values, similarly for the bond angles (as listed in Table 1 ▸). For example, the *trans* P1—Ru1—P2 angle, 173.08 (5)°, exhibits a distortion of about 7° from linearity. The phosphane ligands coordinate through the following bite angles, 82.87 (4) and 82.54 (4)° for Br1—Ru1—P2 and Br2—Ru1—P1, respectively. Thus, these bond lengths and angles are in good match with those of other independent mol­ecules within the asymmetric unit of this complex. Meanwhile, as at the time of this report, there is no complex of similar structural arrangement to make comparison with the title complex herein. The crystal packing arrangement (Fig. 2 ▸) exhibits a twofold rotational axis along [010], an inversion centre at origin and a glide plane perpendicular to [010] with the glide component along the direction [1/2 0 1/2]. The twofold rotation axis passes through the Ru3 central atom of one of the complex mol­ecules. The stability of the packing is influenced by hydrogen bonding of the type O—H⋯*X* (where *X* = O or Cl) and non-classical hydrogen bonding inter­actions (Table 2 ▸). The solvent methanol mol­ecules are enclosed in an irregular void of volume 643 Å^3^.

## Synthesis and crystallization

(2-Bromo­phen­yl)di­cyclo­hexyl­phosphine (28 mg, 0.08 mmol, 2 eq) was added to a solution of [*η*^6^-(C_6_H_6_)_2_RuCl_2_]_2_ (20 mg, 0.04 mmol, 1 eq) in methanol (1 ml) followed by continuous stirring at room temperature for 24 h. Yellow crystals of the title complex were obtained by slow evaporation of the solvent at room temperature.

## Refinement

A summary of the crystal data, data collection and structure refinement details is provided in Table 3 ▸.

## Supplementary Material

Crystal structure: contains datablock(s) I. DOI: 10.1107/S2414314626010862/bh4108sup1.cif

Structure factors: contains datablock(s) I. DOI: 10.1107/S2414314626010862/bh4108Isup2.hkl

CCDC reference: 2596337

Additional supporting information:  crystallographic information; 3D view; checkCIF report

## Figures and Tables

**Figure 1:**
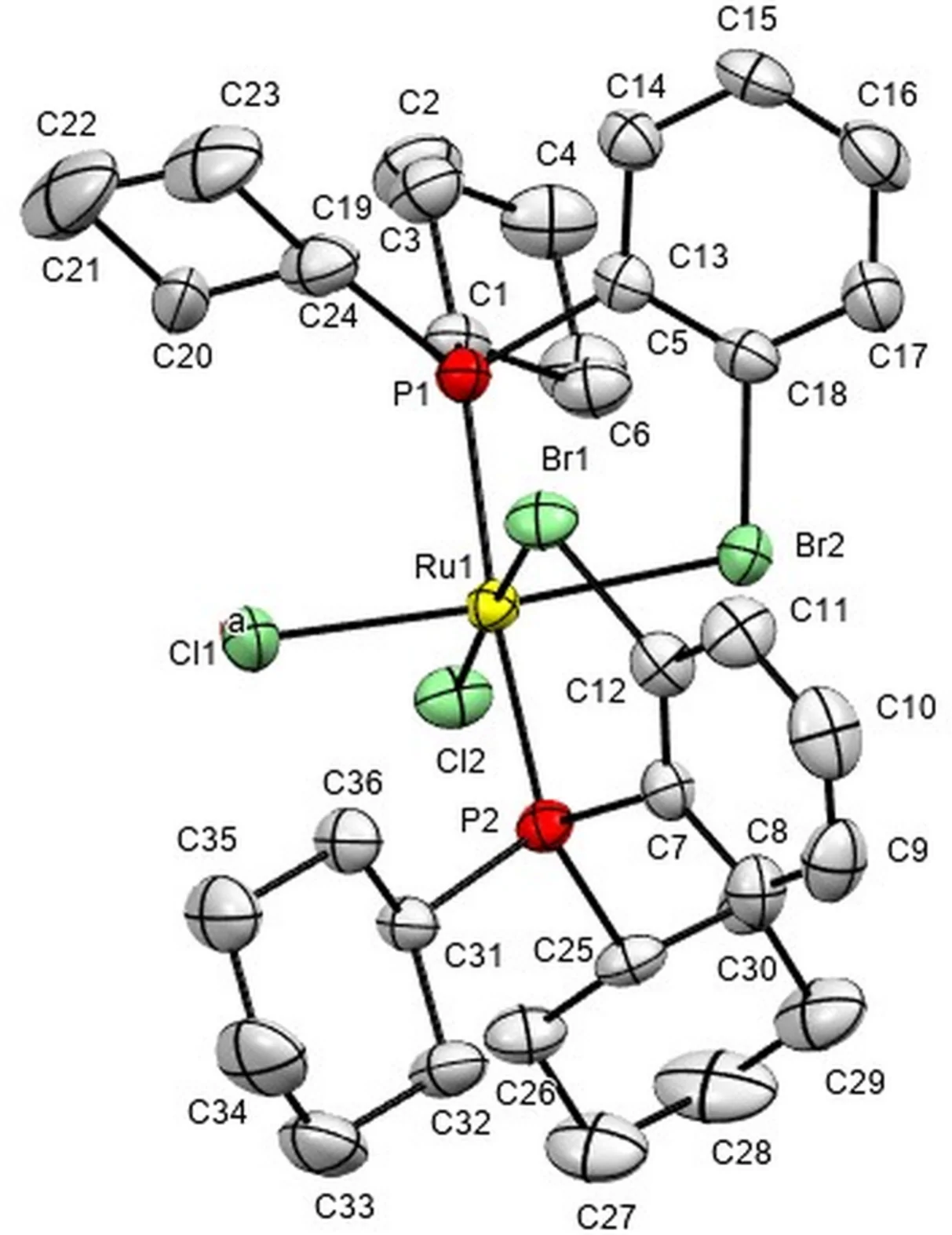
Mol­ecular structure of the title complex with displacement ellipsoids shown at the 50% probability level (hydrogen atoms are omitted for clarity). Only the mol­ecule based on Ru1 in the asymmetric unit is shown, and the methanol solvent is omitted.

**Figure 2:**
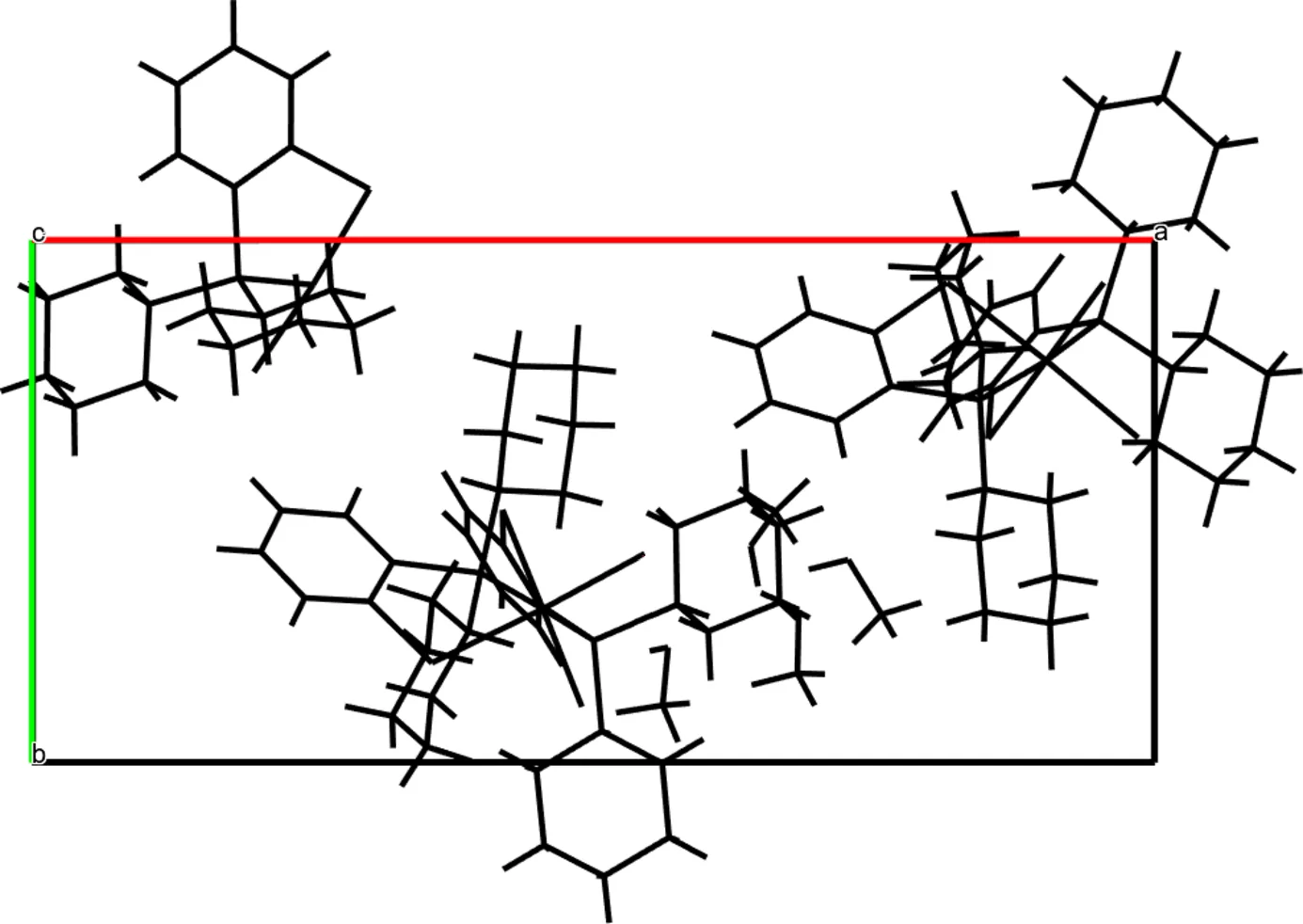
The molecules in the asymmetric unit, plus the bonds required to complete the molecular units.

**Table 1:** Selected bond angles (°)

P2—Ru1—P1	173.08 (5)	P2—Ru1—Br1	82.87 (4)
P2—Ru1—Cl1	90.86 (5)	P1—Ru1—Br1	91.73 (4)
P1—Ru1—Cl1	93.82 (5)	Cl1—Ru1—Br1	93.73 (4)
P2—Ru1—Cl2	94.63 (5)	Cl2—Ru1—Br1	177.22 (4)
P1—Ru1—Cl2	90.67 (5)	P2—Ru1—Br2	92.70 (4)
Cl1—Ru1—Cl2	87.48 (5)	P1—Ru1—Br2	82.54 (4)

**Table 2:** Hydrogen-bond geometry (Å, °)

*D*—H⋯*A*	*D*—H	H⋯*A*	*D*⋯*A*	*D*—H⋯*A*
O1—H1⋯Cl2	0.82	2.47	3.243 (6)	157
O2—H2⋯O1	0.82	2.29	2.731 (10)	114
O3—H3⋯Cl1	0.82	2.44	3.259 (7)	172
O4—H4⋯O2	0.82	2.42	2.735 (12)	104
C6—H6*B*⋯Cl2	0.97	2.70	3.453 (6)	135
C11—H11⋯Cl5^i^	0.93	2.73	3.474 (6)	138
C20—H20*B*⋯Cl1	0.97	2.67	3.511 (6)	146

Symmetry code: (i) 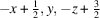.

**Table 3:** Experimental details

Crystal data	
Chemical formula	[RuCl_2_(C_18_H_26_BrP)_2_]·1.6CH_4_O
*M*_r_	929.77
Crystal system, space group	Monoclinic, *P*2/*n*
Temperature (K)	273
*a*, *b*, *c* (Å)	22.3929 (8), 10.2987 (3), 44.7559 (16)
β (°)	98.837 (1)
*V* (Å^3^)	10199.0 (6)
*Z*	10
Radiation type	Mo *K*α
μ (mm^−1^)	2.58
Crystal size (mm)	0.24 × 0.15 × 0.13

Data collection	
Diffractometer	Bruker APEXII CCD
Absorption correction	Multi-scan (*SADABS*; Krause *et al.*, 2015 ▸)
*T*_min_, *T*_max_	0.608, 0.746
No. of measured, independent and observed [*I* > 2σ(*I*)] reflections	199859, 25316, 14549
*R*_int_	0.156
(sin θ/λ)_max_ (Å^−1^)	0.667

Refinement	
*R*[*F*^2^ > 2σ(*F*^2^)], *wR*(*F*^2^), *S*	0.056, 0.127, 1.02
No. of reflections	25316
No. of parameters	1050
H-atom treatment	H-atom parameters constrained
Δρ_max_, Δρ_min_ (e Å^−3^)	1.17, −1.29

Computer programs: *APEX2* and *SAINT* (Bruker, 2010 ▸), *SHELXT* (Sheldrick, 2015*a* ▸), *SHELXL* (Sheldrick, 2015*b* ▸), *ORTEP-3 for Windows* (Farrugia, 2012 ▸), *Mercury* (Macrae *et al.*, 2020 ▸) and *publCIF* (Westrip, 2010 ▸).

## full crystallographic data

### Bis[(2-bromophenyl)dicyclohexylphosphane-κ2Br,P]dichloridoruthenium(II) methanol 1.6-solvate Crystal data

**Table:**

[RuCl_2_(C_18_H_26_BrP)_2_]·1.6CH_4_O	*F*(000) = 4748
*M**_r_* = 929.77	*D*_x_ = 1.514 Mg m^−^^3^
Monoclinic, *P*2/*n*	Mo *K*α radiation, λ = 0.71073 Å
*a* = 22.3929 (8) Å	Cell parameters from 7366 reflections
*b* = 10.2987 (3) Å	θ = 2.2–22.4°
*c* = 44.7559 (16) Å	µ = 2.58 mm^−^^1^
β = 98.837 (1)°	*T* = 273 K
*V* = 10199.0 (6) Å^3^	Block, orange
*Z* = 10	0.24 × 0.15 × 0.13 mm

### Bis[(2-bromophenyl)dicyclohexylphosphane-κ2Br,P]dichloridoruthenium(II) methanol 1.6-solvate Data collection

**Table:**

Bruker APEXII CCD diffractometer	25316 independent reflections
Radiation source: sealed tube	14549 reflections with *I* > 2σ(*I*)
Detector resolution: 8.3333 pixels mm^-1^	*R*_int_ = 0.156
φ and ω scans	θ_max_ = 28.3°, θ_min_ = 1.5°
Absorption correction: multi-scan (SADABS; Krause *et al.*, 2015)	*h* = −29→29
*T*_min_ = 0.608, *T*_max_ = 0.746	*k* = −13→13
199859 measured reflections	*l* = −57→59

### Bis[(2-bromophenyl)dicyclohexylphosphane-κ2Br,P]dichloridoruthenium(II) methanol 1.6-solvate Refinement

**Table:**

Refinement on *F*^2^	Secondary atom site location: difference Fourier map
Least-squares matrix: full	Hydrogen site location: inferred from neighbouring sites
*R*[*F*^2^ > 2σ(*F*^2^)] = 0.056	H-atom parameters constrained
*wR*(*F*^2^) = 0.127	*w* = 1/[σ^2^(*F*_o_^2^) + (0.0383*P*)^2^ + 9.2315*P*] where *P* = (*F*_o_^2^ + 2*F*_c_^2^)/3
*S* = 1.02	(Δ/σ)_max_ = 0.003
25316 reflections	Δρ_max_ = 1.17 e Å^−^^3^
1050 parameters	Δρ_min_ = −1.29 e Å^−^^3^
0 restraints	Extinction correction: SHELXL (Sheldrick, 2015*b*), Fc^*^=kFc[1+0.001xFc^2^λ^3^/sin(2θ)]^-1/4^
Primary atom site location: dual	Extinction coefficient: 0.00025 (3)

### Bis[(2-bromophenyl)dicyclohexylphosphane-κ2Br,P]dichloridoruthenium(II) methanol 1.6-solvate Fractional atomic coordinates and isotropic or equivalent isotropic displacement parameters (Å^2^)

**Table:**

	*x*	*y*	*z*	*U*_iso_*/*U*_eq_	
Ru1	0.45503 (2)	0.70639 (4)	0.62567 (2)	0.02724 (10)	
Ru2	0.90381 (2)	0.23092 (4)	0.60624 (2)	0.02968 (11)	
Ru3	0.250000	0.08549 (6)	0.750000	0.03808 (16)	
Br1	0.41905 (3)	0.51668 (5)	0.65268 (2)	0.03843 (14)	
Br2	0.35608 (2)	0.80978 (5)	0.63161 (2)	0.03800 (14)	
Br3	0.81523 (3)	0.08129 (5)	0.59229 (2)	0.04178 (15)	
Br4	0.85176 (3)	0.38066 (5)	0.56601 (2)	0.03921 (15)	
Br5	0.30047 (3)	−0.09785 (6)	0.72561 (2)	0.04730 (16)	
Cl1	0.54584 (6)	0.60056 (14)	0.61726 (3)	0.0448 (4)	
Cl2	0.48976 (7)	0.89386 (13)	0.60203 (3)	0.0426 (3)	
Cl3	0.95502 (7)	0.08131 (13)	0.64207 (3)	0.0417 (3)	
Cl4	0.98566 (7)	0.37863 (13)	0.62006 (3)	0.0435 (4)	
Cl5	0.19731 (8)	0.25278 (16)	0.77183 (4)	0.0622 (5)	
P1	0.39951 (6)	0.63740 (13)	0.57873 (3)	0.0306 (3)	
P2	0.50036 (6)	0.76683 (12)	0.67528 (3)	0.0288 (3)	
P3	0.95116 (6)	0.15427 (12)	0.56622 (3)	0.0287 (3)	
P4	0.84465 (7)	0.30566 (13)	0.64276 (3)	0.0330 (3)	
P5	0.18352 (7)	0.07226 (14)	0.70276 (3)	0.0378 (4)	
O1	0.5666 (4)	0.7798 (7)	0.55210 (18)	0.149 (3)	
H1	0.550219	0.787057	0.567178	0.224*	
O2	0.6402 (4)	0.5856 (7)	0.53765 (16)	0.126 (2)	
H2	0.646819	0.663398	0.540235	0.189*	
O3	0.6831 (3)	0.7079 (10)	0.6371 (2)	0.170 (3)	
H3	0.649325	0.675398	0.633465	0.255*	
O4	0.7272 (4)	0.6120 (8)	0.5014 (2)	0.164 (3)	
H4	0.691889	0.630702	0.495053	0.245*	
C1	0.3888 (2)	0.7500 (5)	0.54567 (12)	0.0347 (13)	
H1A	0.429133	0.776049	0.542076	0.042*	
C2	0.3565 (3)	0.6905 (6)	0.51601 (13)	0.0487 (16)	
H2A	0.378430	0.614170	0.511112	0.058*	
H2B	0.316154	0.663409	0.518692	0.058*	
C3	0.3521 (3)	0.7869 (7)	0.49000 (14)	0.064 (2)	
H3A	0.392483	0.807348	0.486049	0.077*	
H3B	0.330097	0.747460	0.471924	0.077*	
C4	0.3207 (3)	0.9112 (6)	0.49680 (14)	0.0590 (19)	
H4A	0.278731	0.892828	0.498276	0.071*	
H4B	0.321561	0.972531	0.480435	0.071*	
C5	0.3514 (3)	0.9701 (6)	0.52617 (14)	0.0535 (17)	
H5A	0.328942	1.046100	0.530833	0.064*	
H5B	0.391744	0.998109	0.523738	0.064*	
C6	0.3557 (3)	0.8741 (5)	0.55250 (13)	0.0436 (15)	
H6A	0.315365	0.852321	0.556230	0.052*	
H6B	0.377131	0.914454	0.570625	0.052*	
C7	0.4518 (2)	0.6973 (5)	0.70083 (12)	0.0331 (12)	
C8	0.4510 (2)	0.7410 (5)	0.73039 (13)	0.0406 (14)	
H8	0.472478	0.815312	0.737213	0.049*	
C9	0.4186 (3)	0.6748 (6)	0.74965 (14)	0.0514 (16)	
H9	0.418133	0.705871	0.769122	0.062*	
C10	0.3874 (3)	0.5645 (6)	0.74028 (15)	0.0549 (18)	
H10	0.366098	0.520622	0.753422	0.066*	
C11	0.3875 (3)	0.5181 (5)	0.71140 (14)	0.0447 (15)	
H11	0.366251	0.443249	0.704735	0.054*	
C12	0.4197 (2)	0.5856 (5)	0.69277 (12)	0.0363 (13)	
C13	0.3212 (2)	0.6168 (5)	0.58611 (12)	0.0309 (12)	
C14	0.2798 (2)	0.5276 (5)	0.57109 (13)	0.0386 (14)	
H14	0.291481	0.474357	0.556239	0.046*	
C15	0.2218 (3)	0.5174 (6)	0.57795 (14)	0.0457 (15)	
H15	0.195128	0.456705	0.567977	0.055*	
C16	0.2035 (3)	0.5970 (6)	0.59952 (15)	0.0566 (18)	
H16	0.164149	0.591121	0.603637	0.068*	
C17	0.2427 (3)	0.6852 (6)	0.61507 (14)	0.0478 (15)	
H17	0.230678	0.738451	0.629833	0.057*	
C18	0.3005 (2)	0.6920 (5)	0.60804 (12)	0.0358 (13)	
C19	0.4158 (3)	0.4789 (5)	0.56218 (13)	0.0395 (14)	
H19	0.381884	0.458337	0.546329	0.047*	
C20	0.4732 (3)	0.4849 (6)	0.54722 (14)	0.0568 (18)	
H20A	0.469219	0.552989	0.532055	0.068*	
H20B	0.507984	0.504483	0.562289	0.068*	
C21	0.4820 (4)	0.3535 (8)	0.53245 (18)	0.086 (3)	
H21A	0.449060	0.339149	0.515999	0.103*	
H21B	0.519361	0.355107	0.523995	0.103*	
C22	0.4841 (4)	0.2439 (8)	0.5545 (2)	0.103 (4)	
H22A	0.520184	0.252008	0.569434	0.124*	
H22B	0.486629	0.162427	0.543891	0.124*	
C23	0.4291 (4)	0.2410 (6)	0.57051 (18)	0.080 (3)	
H23A	0.393428	0.221287	0.556024	0.096*	
H23B	0.433972	0.173052	0.585705	0.096*	
C24	0.4208 (3)	0.3697 (5)	0.58536 (14)	0.0525 (17)	
H24A	0.454827	0.385897	0.601104	0.063*	
H24B	0.384418	0.367173	0.594663	0.063*	
C25	0.5074 (2)	0.9407 (4)	0.68529 (12)	0.0351 (13)	
H25	0.514951	0.946618	0.707400	0.042*	
C26	0.5614 (3)	1.0021 (5)	0.67351 (14)	0.0476 (16)	
H26A	0.598052	0.955925	0.681678	0.057*	
H26B	0.556035	0.995224	0.651648	0.057*	
C27	0.5675 (3)	1.1442 (5)	0.68268 (17)	0.065 (2)	
H27A	0.600960	1.182748	0.674410	0.078*	
H27B	0.576171	1.150618	0.704535	0.078*	
C28	0.5096 (4)	1.2181 (6)	0.67121 (19)	0.085 (3)	
H28A	0.514132	1.307870	0.677747	0.102*	
H28B	0.502701	1.217109	0.649275	0.102*	
C29	0.4561 (3)	1.1593 (6)	0.68275 (17)	0.066 (2)	
H29A	0.419736	1.205642	0.674242	0.080*	
H29B	0.461143	1.168208	0.704573	0.080*	
C30	0.4493 (3)	1.0158 (5)	0.67428 (13)	0.0413 (14)	
H30A	0.439430	1.007654	0.652492	0.050*	
H30B	0.416365	0.978711	0.683207	0.050*	
C31	0.5747 (2)	0.6983 (5)	0.69182 (12)	0.0335 (12)	
H31	0.603071	0.721287	0.678016	0.040*	
C32	0.6024 (3)	0.7494 (5)	0.72326 (13)	0.0467 (16)	
H32A	0.604577	0.843393	0.722686	0.056*	
H32B	0.576538	0.725448	0.737875	0.056*	
C33	0.6660 (3)	0.6939 (7)	0.73327 (16)	0.067 (2)	
H33A	0.692828	0.723712	0.719639	0.080*	
H33B	0.681604	0.724829	0.753425	0.080*	
C34	0.6643 (3)	0.5465 (7)	0.73325 (17)	0.076 (2)	
H34A	0.705020	0.513019	0.738434	0.091*	
H34B	0.640894	0.516711	0.748472	0.091*	
C35	0.6366 (3)	0.4946 (6)	0.70281 (16)	0.0617 (19)	
H35A	0.634479	0.400625	0.703693	0.074*	
H35B	0.661906	0.517693	0.687890	0.074*	
C36	0.5736 (2)	0.5493 (5)	0.69330 (13)	0.0407 (14)	
H36A	0.547663	0.522141	0.707648	0.049*	
H36B	0.556819	0.514883	0.673615	0.049*	
C37	1.0163 (2)	0.2463 (4)	0.55505 (11)	0.0280 (11)	
H37	1.047546	0.250198	0.572942	0.034*	
C38	1.0452 (2)	0.1830 (5)	0.52985 (12)	0.0380 (13)	
H38A	1.056036	0.094136	0.535431	0.046*	
H38B	1.016035	0.181039	0.511430	0.046*	
C39	1.1013 (3)	0.2561 (5)	0.52403 (14)	0.0433 (15)	
H39A	1.131946	0.250514	0.541816	0.052*	
H39B	1.117213	0.215437	0.507365	0.052*	
C40	1.0879 (3)	0.3980 (5)	0.51654 (13)	0.0450 (15)	
H40A	1.062144	0.404486	0.497082	0.054*	
H40B	1.125347	0.443355	0.515157	0.054*	
C41	1.0566 (2)	0.4616 (5)	0.54077 (13)	0.0415 (14)	
H41A	1.045455	0.549783	0.534710	0.050*	
H41B	1.084589	0.465503	0.559590	0.050*	
C42	1.0002 (2)	0.3868 (5)	0.54584 (12)	0.0361 (13)	
H42A	0.971093	0.387412	0.527416	0.043*	
H42B	0.981945	0.428867	0.561588	0.043*	
C43	0.7653 (3)	0.2795 (5)	0.62603 (12)	0.0394 (14)	
C44	0.7165 (3)	0.3466 (6)	0.63470 (14)	0.0526 (17)	
H44	0.723657	0.417168	0.647718	0.063*	
C45	0.6579 (3)	0.3098 (7)	0.62424 (16)	0.064 (2)	
H45	0.626143	0.357091	0.629961	0.076*	
C46	0.6456 (3)	0.2048 (8)	0.60556 (17)	0.071 (2)	
H46	0.605821	0.178452	0.599540	0.085*	
C47	0.6922 (3)	0.1382 (7)	0.59569 (14)	0.0539 (17)	
H47	0.684541	0.068475	0.582446	0.065*	
C48	0.7508 (3)	0.1776 (5)	0.60598 (13)	0.0394 (14)	
C49	0.8951 (2)	0.1760 (5)	0.53185 (12)	0.0322 (12)	
C50	0.8930 (3)	0.1001 (5)	0.50594 (12)	0.0400 (14)	
H50	0.918304	0.028245	0.506369	0.048*	
C51	0.8544 (3)	0.1285 (6)	0.47966 (14)	0.0492 (16)	
H51	0.853461	0.075798	0.462726	0.059*	
C52	0.8172 (3)	0.2359 (6)	0.47865 (14)	0.0481 (16)	
H52	0.792012	0.256763	0.460775	0.058*	
C53	0.8169 (3)	0.3129 (6)	0.50411 (13)	0.0448 (15)	
H53	0.791804	0.384995	0.503671	0.054*	
C54	0.8552 (2)	0.2788 (5)	0.53002 (12)	0.0358 (13)	
C55	0.9759 (2)	−0.0171 (4)	0.56493 (12)	0.0314 (12)	
H55	0.983899	−0.035160	0.544407	0.038*	
C56	1.0351 (2)	−0.0396 (5)	0.58695 (13)	0.0391 (14)	
H56A	1.066275	0.017793	0.581706	0.047*	
H56B	1.028771	−0.018953	0.607374	0.047*	
C57	1.0559 (3)	−0.1796 (5)	0.58569 (15)	0.0530 (17)	
H57A	1.065531	−0.197948	0.565701	0.064*	
H57B	1.092405	−0.192012	0.600188	0.064*	
C58	1.0078 (3)	−0.2734 (5)	0.59266 (15)	0.060 (2)	
H58A	1.021633	−0.361816	0.590808	0.073*	
H58B	1.000772	−0.260502	0.613306	0.073*	
C59	0.9501 (3)	−0.2531 (5)	0.57152 (16)	0.0576 (19)	
H59A	0.919513	−0.311108	0.577100	0.069*	
H59B	0.956331	−0.275226	0.551157	0.069*	
C60	0.9271 (3)	−0.1117 (5)	0.57190 (14)	0.0453 (15)	
H60A	0.891143	−0.101681	0.556960	0.054*	
H60B	0.916476	−0.092034	0.591616	0.054*	
C61	0.8488 (3)	0.4769 (5)	0.65511 (13)	0.0391 (14)	
H61	0.814780	0.493268	0.665987	0.047*	
C62	0.9066 (3)	0.5015 (5)	0.67688 (13)	0.0431 (15)	
H62A	0.941064	0.480711	0.666991	0.052*	
H62B	0.907792	0.444757	0.694264	0.052*	
C63	0.9114 (3)	0.6434 (6)	0.68758 (15)	0.0555 (18)	
H63A	0.879031	0.662304	0.699078	0.067*	
H63B	0.949555	0.656698	0.700697	0.067*	
C64	0.9071 (4)	0.7338 (6)	0.66079 (17)	0.070 (2)	
H64A	0.941361	0.719640	0.650325	0.084*	
H64B	0.908540	0.823041	0.667817	0.084*	
C65	0.8491 (4)	0.7119 (6)	0.63912 (17)	0.069 (2)	
H65A	0.848268	0.768972	0.621824	0.083*	
H65B	0.814910	0.733390	0.649099	0.083*	
C66	0.8436 (3)	0.5723 (5)	0.62837 (14)	0.0508 (16)	
H66A	0.875143	0.553785	0.616357	0.061*	
H66B	0.804907	0.560167	0.615591	0.061*	
C67	0.8468 (3)	0.2122 (5)	0.67850 (12)	0.0352 (13)	
H67	0.889434	0.207109	0.687567	0.042*	
C68	0.8252 (3)	0.0718 (5)	0.67327 (13)	0.0440 (15)	
H68A	0.782275	0.071372	0.665560	0.053*	
H68B	0.846103	0.031975	0.658170	0.053*	
C69	0.8367 (3)	−0.0069 (6)	0.70208 (14)	0.0538 (17)	
H69A	0.879851	−0.012669	0.708933	0.065*	
H69B	0.821289	−0.094295	0.698142	0.065*	
C70	0.8063 (3)	0.0548 (6)	0.72657 (15)	0.063 (2)	
H70A	0.817030	0.006711	0.745260	0.075*	
H70B	0.762766	0.049734	0.720815	0.075*	
C71	0.8247 (3)	0.1962 (6)	0.73179 (14)	0.0552 (17)	
H71A	0.801829	0.234402	0.746279	0.066*	
H71B	0.867262	0.200568	0.740233	0.066*	
C72	0.8137 (3)	0.2733 (5)	0.70247 (13)	0.0452 (15)	
H72A	0.827578	0.361768	0.706294	0.054*	
H72B	0.770651	0.275872	0.695018	0.054*	
C73	0.1041 (2)	0.1225 (5)	0.70131 (14)	0.0408 (14)	
H73	0.080878	0.091360	0.682300	0.049*	
C74	0.0769 (3)	0.0631 (6)	0.72773 (13)	0.0460 (15)	
H74A	0.076090	−0.030727	0.725817	0.055*	
H74B	0.102138	0.085016	0.746656	0.055*	
C75	0.0134 (3)	0.1126 (6)	0.72822 (14)	0.0492 (16)	
H75A	−0.002365	0.075115	0.745319	0.059*	
H75B	−0.012586	0.085544	0.709904	0.059*	
C76	0.0126 (3)	0.2598 (6)	0.73057 (14)	0.0550 (17)	
H76A	−0.028420	0.289524	0.730777	0.066*	
H76B	0.037109	0.286888	0.749309	0.066*	
C77	0.0370 (3)	0.3197 (6)	0.70413 (14)	0.0515 (16)	
H77A	0.037616	0.413376	0.706312	0.062*	
H77B	0.010280	0.298484	0.685595	0.062*	
C78	0.1006 (3)	0.2719 (5)	0.70180 (14)	0.0477 (16)	
H78A	0.128501	0.304510	0.718882	0.057*	
H78B	0.112897	0.306117	0.683494	0.057*	
C79	0.1799 (3)	−0.1020 (5)	0.69294 (12)	0.0400 (14)	
C80	0.1296 (3)	−0.1648 (6)	0.67696 (13)	0.0460 (15)	
H80	0.095674	−0.116417	0.669219	0.055*	
C81	0.1293 (3)	−0.2985 (6)	0.67239 (15)	0.0592 (18)	
H81	0.095059	−0.338832	0.662092	0.071*	
C82	0.1793 (4)	−0.3705 (6)	0.68302 (16)	0.064 (2)	
H82	0.179037	−0.459867	0.679975	0.077*	
C83	0.2306 (3)	−0.3103 (6)	0.69840 (15)	0.0564 (18)	
H83	0.264852	−0.358863	0.705500	0.068*	
C84	0.2303 (3)	−0.1794 (5)	0.70298 (13)	0.0456 (15)	
C85	0.2054 (3)	0.1458 (6)	0.66798 (13)	0.0461 (15)	
H85	0.210586	0.238956	0.672016	0.055*	
C86	0.1574 (3)	0.1332 (7)	0.63991 (14)	0.064 (2)	
H86A	0.119487	0.169205	0.644062	0.077*	
H86B	0.150969	0.042262	0.634798	0.077*	
C87	0.1777 (3)	0.2058 (8)	0.61310 (15)	0.078 (2)	
H87A	0.147413	0.195205	0.595315	0.093*	
H87B	0.181193	0.297807	0.617733	0.093*	
C88	0.2386 (3)	0.1552 (8)	0.60633 (16)	0.076 (2)	
H88A	0.234489	0.065171	0.599967	0.091*	
H88B	0.251116	0.205261	0.590016	0.091*	
C89	0.2852 (3)	0.1662 (7)	0.63406 (15)	0.0637 (19)	
H89A	0.291982	0.257180	0.639068	0.076*	
H89B	0.323029	0.129892	0.629859	0.076*	
C90	0.2662 (3)	0.0956 (6)	0.66117 (13)	0.0493 (16)	
H90A	0.296785	0.108292	0.678764	0.059*	
H90B	0.263159	0.003250	0.656964	0.059*	
C91	0.5618 (5)	0.8909 (8)	0.5368 (2)	0.110 (3)	
H91A	0.585947	0.886844	0.520891	0.166*	
H91B	0.575582	0.961117	0.550236	0.166*	
H91C	0.520273	0.905101	0.528292	0.166*	
C92	0.6620 (5)	0.5203 (10)	0.5631 (2)	0.140 (5)	
H92A	0.629606	0.475535	0.570473	0.211*	
H92B	0.680399	0.580351	0.578194	0.211*	
H92C	0.691675	0.458517	0.558815	0.211*	
C93	0.6819 (6)	0.8298 (9)	0.6247 (4)	0.200 (8)	
H93A	0.697253	0.891404	0.640104	0.301*	
H93B	0.641033	0.852103	0.616481	0.301*	
H93C	0.706461	0.831257	0.608959	0.301*	
C94	0.7569 (6)	0.7166 (11)	0.5104 (4)	0.214 (9)	
H94A	0.768626	0.760530	0.493278	0.321*	
H94B	0.792378	0.693808	0.524315	0.321*	
H94C	0.731795	0.772690	0.520238	0.321*	

### Bis[(2-bromophenyl)dicyclohexylphosphane-κ2Br,P]dichloridoruthenium(II) methanol 1.6-solvate Atomic displacement parameters (Å^2^)

**Table:**

	*U*^11^	*U*^22^	*U*^33^	*U*^12^	*U*^13^	*U*^23^
Ru1	0.0284 (2)	0.0241 (2)	0.0287 (2)	0.00062 (17)	0.00281 (18)	−0.00126 (17)
Ru2	0.0351 (3)	0.0267 (2)	0.0288 (2)	0.00205 (19)	0.00984 (19)	0.00085 (17)
Ru3	0.0364 (4)	0.0388 (4)	0.0381 (4)	0.000	0.0028 (3)	0.000
Br1	0.0462 (3)	0.0264 (3)	0.0411 (3)	−0.0056 (2)	0.0017 (3)	0.0015 (2)
Br2	0.0356 (3)	0.0359 (3)	0.0423 (3)	0.0054 (2)	0.0052 (3)	−0.0081 (2)
Br3	0.0428 (3)	0.0393 (3)	0.0455 (4)	−0.0035 (3)	0.0139 (3)	−0.0065 (3)
Br4	0.0446 (4)	0.0350 (3)	0.0388 (3)	0.0106 (3)	0.0090 (3)	0.0019 (2)
Br5	0.0404 (4)	0.0535 (4)	0.0470 (4)	0.0102 (3)	0.0036 (3)	0.0007 (3)
Cl1	0.0342 (8)	0.0550 (9)	0.0445 (9)	0.0093 (7)	0.0034 (7)	−0.0103 (7)
Cl2	0.0546 (9)	0.0374 (7)	0.0355 (8)	−0.0108 (7)	0.0056 (7)	0.0035 (6)
Cl3	0.0513 (9)	0.0413 (8)	0.0338 (8)	0.0104 (7)	0.0105 (7)	0.0071 (6)
Cl4	0.0502 (9)	0.0411 (8)	0.0409 (9)	−0.0089 (7)	0.0127 (7)	−0.0065 (6)
Cl5	0.0656 (11)	0.0611 (10)	0.0575 (11)	0.0234 (9)	0.0012 (9)	−0.0121 (8)
P1	0.0293 (8)	0.0296 (7)	0.0319 (8)	0.0022 (6)	0.0020 (6)	−0.0028 (6)
P2	0.0326 (8)	0.0228 (6)	0.0305 (8)	0.0000 (6)	0.0038 (6)	−0.0008 (5)
P3	0.0326 (8)	0.0259 (7)	0.0289 (8)	0.0025 (6)	0.0085 (6)	0.0004 (6)
P4	0.0384 (8)	0.0296 (7)	0.0332 (8)	0.0047 (6)	0.0127 (7)	0.0013 (6)
P5	0.0323 (8)	0.0405 (8)	0.0396 (9)	0.0015 (7)	0.0026 (7)	0.0047 (7)
O1	0.205 (8)	0.144 (6)	0.124 (7)	0.078 (6)	0.102 (6)	0.054 (5)
O2	0.130 (6)	0.155 (6)	0.103 (5)	0.032 (6)	0.045 (5)	0.013 (5)
O3	0.081 (5)	0.223 (10)	0.203 (9)	−0.014 (6)	0.012 (6)	−0.017 (8)
O4	0.198 (9)	0.163 (8)	0.147 (8)	−0.030 (7)	0.082 (8)	−0.040 (6)
C1	0.035 (3)	0.037 (3)	0.031 (3)	−0.005 (2)	0.002 (2)	0.001 (2)
C2	0.058 (4)	0.045 (3)	0.040 (4)	0.004 (3)	−0.001 (3)	−0.006 (3)
C3	0.080 (5)	0.078 (5)	0.032 (4)	0.003 (4)	−0.001 (4)	0.001 (3)
C4	0.075 (5)	0.057 (4)	0.041 (4)	−0.011 (4)	−0.003 (4)	0.015 (3)
C5	0.065 (4)	0.040 (3)	0.052 (4)	−0.004 (3)	−0.001 (3)	0.009 (3)
C6	0.052 (4)	0.039 (3)	0.036 (3)	−0.003 (3)	−0.004 (3)	0.002 (3)
C7	0.030 (3)	0.033 (3)	0.036 (3)	0.004 (2)	0.005 (2)	0.002 (2)
C8	0.039 (3)	0.042 (3)	0.042 (4)	0.005 (3)	0.009 (3)	0.002 (3)
C9	0.060 (4)	0.060 (4)	0.037 (4)	0.011 (3)	0.017 (3)	0.005 (3)
C10	0.056 (4)	0.056 (4)	0.058 (5)	0.004 (3)	0.023 (4)	0.026 (3)
C11	0.055 (4)	0.035 (3)	0.045 (4)	−0.005 (3)	0.011 (3)	0.008 (3)
C12	0.036 (3)	0.037 (3)	0.035 (3)	0.001 (3)	0.004 (3)	0.008 (2)
C13	0.032 (3)	0.025 (3)	0.034 (3)	0.002 (2)	0.001 (2)	0.006 (2)
C14	0.034 (3)	0.038 (3)	0.043 (4)	0.001 (3)	0.003 (3)	0.002 (3)
C15	0.033 (3)	0.047 (4)	0.053 (4)	−0.012 (3)	−0.005 (3)	0.002 (3)
C16	0.032 (4)	0.076 (5)	0.062 (5)	−0.008 (3)	0.009 (3)	0.002 (4)
C17	0.037 (4)	0.056 (4)	0.052 (4)	0.003 (3)	0.011 (3)	−0.002 (3)
C18	0.030 (3)	0.034 (3)	0.041 (3)	−0.002 (2)	−0.003 (3)	0.002 (3)
C19	0.041 (3)	0.035 (3)	0.040 (4)	0.003 (3)	−0.002 (3)	−0.015 (3)
C20	0.041 (4)	0.078 (5)	0.050 (4)	0.016 (3)	0.001 (3)	−0.027 (4)
C21	0.077 (6)	0.106 (7)	0.071 (6)	0.040 (5)	−0.006 (4)	−0.042 (5)
C22	0.120 (8)	0.072 (6)	0.103 (7)	0.056 (6)	−0.031 (6)	−0.046 (5)
C23	0.101 (7)	0.035 (4)	0.094 (6)	0.020 (4)	−0.021 (5)	−0.018 (4)
C24	0.058 (4)	0.038 (3)	0.056 (4)	0.002 (3)	−0.009 (3)	−0.009 (3)
C25	0.049 (4)	0.025 (3)	0.029 (3)	−0.004 (2)	0.000 (3)	−0.003 (2)
C26	0.057 (4)	0.037 (3)	0.047 (4)	−0.013 (3)	0.002 (3)	−0.002 (3)
C27	0.076 (5)	0.035 (3)	0.079 (5)	−0.014 (3)	−0.009 (4)	0.007 (3)
C28	0.102 (7)	0.029 (3)	0.108 (7)	−0.012 (4)	−0.034 (5)	0.012 (4)
C29	0.083 (5)	0.033 (3)	0.074 (5)	0.017 (4)	−0.015 (4)	−0.009 (3)
C30	0.050 (4)	0.030 (3)	0.042 (4)	0.009 (3)	0.001 (3)	−0.001 (3)
C31	0.033 (3)	0.030 (3)	0.036 (3)	−0.001 (2)	−0.001 (2)	−0.001 (2)
C32	0.054 (4)	0.040 (3)	0.041 (4)	0.001 (3)	−0.011 (3)	−0.009 (3)
C33	0.051 (4)	0.071 (5)	0.069 (5)	−0.001 (4)	−0.019 (4)	−0.009 (4)
C34	0.058 (5)	0.072 (5)	0.085 (6)	0.020 (4)	−0.028 (4)	−0.002 (4)
C35	0.051 (4)	0.047 (4)	0.081 (5)	0.018 (3)	−0.010 (4)	−0.006 (4)
C36	0.042 (3)	0.031 (3)	0.046 (4)	0.006 (3)	−0.002 (3)	0.003 (3)
C37	0.029 (3)	0.028 (3)	0.027 (3)	0.005 (2)	0.003 (2)	0.006 (2)
C38	0.045 (3)	0.031 (3)	0.041 (3)	0.002 (3)	0.017 (3)	0.000 (2)
C39	0.042 (4)	0.045 (3)	0.047 (4)	0.002 (3)	0.023 (3)	−0.002 (3)
C40	0.048 (4)	0.045 (3)	0.045 (4)	−0.011 (3)	0.019 (3)	0.001 (3)
C41	0.043 (4)	0.032 (3)	0.051 (4)	−0.003 (3)	0.008 (3)	0.001 (3)
C42	0.037 (3)	0.036 (3)	0.035 (3)	0.001 (3)	0.006 (3)	0.002 (2)
C43	0.044 (3)	0.043 (3)	0.033 (3)	0.011 (3)	0.011 (3)	0.012 (3)
C44	0.048 (4)	0.059 (4)	0.052 (4)	0.016 (3)	0.015 (3)	0.007 (3)
C45	0.042 (4)	0.084 (5)	0.067 (5)	0.030 (4)	0.014 (4)	0.009 (4)
C46	0.038 (4)	0.100 (6)	0.072 (5)	0.006 (4)	0.000 (4)	0.005 (5)
C47	0.042 (4)	0.070 (4)	0.048 (4)	−0.004 (3)	0.000 (3)	0.006 (3)
C48	0.036 (3)	0.044 (3)	0.040 (4)	0.004 (3)	0.011 (3)	0.007 (3)
C49	0.030 (3)	0.035 (3)	0.033 (3)	−0.002 (2)	0.009 (2)	0.000 (2)
C50	0.042 (3)	0.043 (3)	0.034 (3)	−0.004 (3)	0.005 (3)	−0.005 (3)
C51	0.050 (4)	0.061 (4)	0.037 (4)	0.001 (3)	0.008 (3)	−0.006 (3)
C52	0.041 (4)	0.065 (4)	0.035 (4)	−0.003 (3)	−0.005 (3)	0.004 (3)
C53	0.037 (3)	0.048 (4)	0.048 (4)	0.005 (3)	−0.001 (3)	0.007 (3)
C54	0.035 (3)	0.035 (3)	0.038 (3)	−0.006 (3)	0.005 (3)	0.001 (2)
C55	0.038 (3)	0.027 (3)	0.031 (3)	0.001 (2)	0.013 (2)	−0.002 (2)
C56	0.044 (4)	0.036 (3)	0.039 (3)	0.006 (3)	0.012 (3)	0.003 (3)
C57	0.068 (5)	0.040 (3)	0.055 (4)	0.019 (3)	0.021 (4)	0.011 (3)
C58	0.099 (6)	0.028 (3)	0.060 (5)	0.017 (3)	0.029 (4)	0.011 (3)
C59	0.087 (5)	0.028 (3)	0.066 (5)	−0.009 (3)	0.036 (4)	−0.002 (3)
C60	0.058 (4)	0.032 (3)	0.049 (4)	−0.007 (3)	0.017 (3)	−0.009 (3)
C61	0.049 (4)	0.029 (3)	0.041 (4)	0.008 (3)	0.014 (3)	0.002 (2)
C62	0.056 (4)	0.036 (3)	0.039 (4)	0.005 (3)	0.012 (3)	−0.006 (3)
C63	0.063 (5)	0.046 (4)	0.060 (5)	−0.003 (3)	0.016 (4)	−0.015 (3)
C64	0.100 (6)	0.034 (4)	0.085 (6)	−0.010 (4)	0.045 (5)	−0.011 (4)
C65	0.112 (7)	0.030 (3)	0.073 (5)	0.016 (4)	0.041 (5)	0.017 (3)
C66	0.066 (4)	0.039 (3)	0.050 (4)	0.011 (3)	0.017 (3)	0.008 (3)
C67	0.043 (3)	0.036 (3)	0.029 (3)	0.000 (3)	0.013 (3)	0.003 (2)
C68	0.058 (4)	0.037 (3)	0.041 (4)	0.000 (3)	0.020 (3)	0.005 (3)
C69	0.067 (5)	0.050 (4)	0.049 (4)	0.011 (3)	0.025 (4)	0.014 (3)
C70	0.089 (5)	0.059 (4)	0.049 (4)	0.007 (4)	0.036 (4)	0.017 (3)
C71	0.059 (4)	0.070 (4)	0.041 (4)	0.010 (4)	0.023 (3)	0.010 (3)
C72	0.053 (4)	0.047 (3)	0.038 (4)	0.002 (3)	0.017 (3)	−0.001 (3)
C73	0.032 (3)	0.042 (3)	0.048 (4)	0.004 (3)	0.006 (3)	0.003 (3)
C74	0.048 (4)	0.045 (3)	0.044 (4)	−0.001 (3)	0.004 (3)	0.005 (3)
C75	0.036 (4)	0.062 (4)	0.050 (4)	−0.001 (3)	0.009 (3)	0.004 (3)
C76	0.049 (4)	0.069 (4)	0.047 (4)	0.014 (3)	0.008 (3)	0.000 (3)
C77	0.045 (4)	0.052 (4)	0.054 (4)	0.014 (3)	−0.001 (3)	−0.002 (3)
C78	0.040 (4)	0.047 (4)	0.053 (4)	0.002 (3)	−0.003 (3)	0.008 (3)
C79	0.046 (4)	0.043 (3)	0.032 (3)	−0.006 (3)	0.008 (3)	0.001 (3)
C80	0.054 (4)	0.047 (4)	0.037 (4)	−0.010 (3)	0.009 (3)	0.001 (3)
C81	0.076 (5)	0.052 (4)	0.049 (4)	−0.022 (4)	0.008 (4)	−0.006 (3)
C82	0.096 (6)	0.041 (4)	0.058 (5)	−0.004 (4)	0.018 (4)	−0.001 (3)
C83	0.069 (5)	0.050 (4)	0.052 (4)	0.007 (4)	0.015 (4)	−0.007 (3)
C84	0.066 (4)	0.036 (3)	0.036 (4)	0.003 (3)	0.012 (3)	−0.003 (3)
C85	0.039 (4)	0.053 (4)	0.046 (4)	−0.002 (3)	0.005 (3)	0.010 (3)
C86	0.046 (4)	0.095 (5)	0.048 (4)	−0.011 (4)	−0.003 (3)	0.020 (4)
C87	0.059 (5)	0.126 (7)	0.045 (4)	−0.011 (5)	−0.003 (4)	0.037 (5)
C88	0.082 (6)	0.101 (6)	0.048 (5)	0.002 (5)	0.017 (4)	0.016 (4)
C89	0.051 (4)	0.082 (5)	0.061 (5)	0.003 (4)	0.017 (4)	0.015 (4)
C90	0.039 (4)	0.067 (4)	0.042 (4)	0.003 (3)	0.006 (3)	0.008 (3)
C91	0.152 (9)	0.080 (6)	0.114 (8)	0.000 (6)	0.067 (7)	0.025 (6)
C92	0.198 (13)	0.141 (10)	0.089 (8)	0.075 (9)	0.042 (8)	0.031 (7)
C93	0.220 (15)	0.051 (6)	0.35 (2)	−0.044 (8)	0.091 (14)	0.037 (9)
C94	0.143 (12)	0.108 (10)	0.42 (3)	−0.042 (9)	0.139 (15)	−0.055 (13)

### Bis[(2-bromophenyl)dicyclohexylphosphane-κ2Br,P]dichloridoruthenium(II) methanol 1.6-solvate Geometric parameters (Å, º)

**Table:**

Ru1—P2	2.3781 (14)	C38—H38B	0.9700
Ru1—P1	2.3795 (15)	C39—C40	1.519 (7)
Ru1—Cl1	2.3873 (14)	C39—H39A	0.9700
Ru1—Cl2	2.3884 (14)	C39—H39B	0.9700
Ru1—Br1	2.4954 (7)	C40—C41	1.526 (7)
Ru1—Br2	2.5091 (7)	C40—H40A	0.9700
Ru2—P3	2.3536 (14)	C40—H40B	0.9700
Ru2—P4	2.3843 (14)	C41—C42	1.526 (7)
Ru2—Cl3	2.3864 (14)	C41—H41A	0.9700
Ru2—Cl4	2.3891 (15)	C41—H41B	0.9700
Ru2—Br3	2.5137 (7)	C42—H42A	0.9700
Ru2—Br4	2.5168 (7)	C42—H42B	0.9700
Ru3—Cl5^i^	2.3811 (16)	C43—C48	1.386 (8)
Ru3—Cl5	2.3811 (16)	C43—C44	1.398 (7)
Ru3—P5^i^	2.3954 (15)	C44—C45	1.376 (9)
Ru3—P5	2.3954 (15)	C44—H44	0.9300
Ru3—Br5	2.5325 (8)	C45—C46	1.369 (9)
Ru3—Br5^i^	2.5325 (8)	C45—H45	0.9300
Br1—C12	1.927 (6)	C46—C47	1.378 (9)
Br2—C18	1.932 (5)	C46—H46	0.9300
Br3—C48	1.927 (5)	C47—C48	1.382 (8)
Br4—C54	1.934 (5)	C47—H47	0.9300
Br5—C84	1.927 (6)	C49—C54	1.380 (7)
P1—C13	1.846 (5)	C49—C50	1.393 (7)
P1—C19	1.852 (5)	C50—C51	1.380 (8)
P1—C1	1.866 (5)	C50—H50	0.9300
P2—C7	1.840 (5)	C51—C52	1.381 (8)
P2—C25	1.847 (5)	C51—H51	0.9300
P2—C31	1.855 (5)	C52—C53	1.389 (8)
P3—C49	1.843 (5)	C52—H52	0.9300
P3—C55	1.854 (5)	C53—C54	1.377 (7)
P3—C37	1.871 (5)	C53—H53	0.9300
P4—C43	1.840 (6)	C55—C60	1.532 (7)
P4—C61	1.847 (5)	C55—C56	1.543 (7)
P4—C67	1.861 (5)	C55—H55	0.9800
P5—C73	1.845 (5)	C56—C57	1.520 (7)
P5—C79	1.847 (6)	C56—H56A	0.9700
P5—C85	1.863 (6)	C56—H56B	0.9700
O1—C91	1.329 (9)	C57—C58	1.515 (8)
O1—H1	0.8200	C57—H57A	0.9700
O2—C92	1.349 (9)	C57—H57B	0.9700
O2—H2	0.8200	C58—C59	1.493 (9)
O3—C93	1.370 (11)	C58—H58A	0.9700
O3—H3	0.8200	C58—H58B	0.9700
O4—C94	1.298 (11)	C59—C60	1.546 (7)
O4—H4	0.8200	C59—H59A	0.9700
C1—C6	1.531 (7)	C59—H59B	0.9700
C1—C2	1.538 (7)	C60—H60A	0.9700
C1—H1A	0.9800	C60—H60B	0.9700
C2—C3	1.522 (8)	C61—C62	1.517 (8)
C2—H2A	0.9700	C61—C66	1.539 (7)
C2—H2B	0.9700	C61—H61	0.9800
C3—C4	1.514 (9)	C62—C63	1.537 (7)
C3—H3A	0.9700	C62—H62A	0.9700
C3—H3B	0.9700	C62—H62B	0.9700
C4—C5	1.514 (8)	C63—C64	1.510 (9)
C4—H4A	0.9700	C63—H63A	0.9700
C4—H4B	0.9700	C63—H63B	0.9700
C5—C6	1.530 (7)	C64—C65	1.513 (10)
C5—H5A	0.9700	C64—H64A	0.9700
C5—H5B	0.9700	C64—H64B	0.9700
C6—H6A	0.9700	C65—C66	1.515 (8)
C6—H6B	0.9700	C65—H65A	0.9700
C7—C12	1.375 (7)	C65—H65B	0.9700
C7—C8	1.400 (7)	C66—H66A	0.9700
C8—C9	1.388 (8)	C66—H66B	0.9700
C8—H8	0.9300	C67—C72	1.530 (7)
C9—C10	1.365 (8)	C67—C68	1.531 (7)
C9—H9	0.9300	C67—H67	0.9800
C10—C11	1.378 (8)	C68—C69	1.511 (7)
C10—H10	0.9300	C68—H68A	0.9700
C11—C12	1.373 (7)	C68—H68B	0.9700
C11—H11	0.9300	C69—C70	1.516 (8)
C13—C18	1.385 (7)	C69—H69A	0.9700
C13—C14	1.402 (7)	C69—H69B	0.9700
C14—C15	1.384 (7)	C70—C71	1.522 (8)
C14—H14	0.9300	C70—H70A	0.9700
C15—C16	1.375 (8)	C70—H70B	0.9700
C15—H15	0.9300	C71—C72	1.521 (8)
C16—C17	1.376 (8)	C71—H71A	0.9700
C16—H16	0.9300	C71—H71B	0.9700
C17—C18	1.378 (7)	C72—H72A	0.9700
C17—H17	0.9300	C72—H72B	0.9700
C19—C24	1.522 (8)	C73—C74	1.538 (7)
C19—C20	1.538 (8)	C73—C78	1.541 (7)
C19—H19	0.9800	C73—H73	0.9800
C20—C21	1.532 (9)	C74—C75	1.514 (7)
C20—H20A	0.9700	C74—H74A	0.9700
C20—H20B	0.9700	C74—H74B	0.9700
C21—C22	1.496 (11)	C75—C76	1.520 (8)
C21—H21A	0.9700	C75—H75A	0.9700
C21—H21B	0.9700	C75—H75B	0.9700
C22—C23	1.517 (11)	C76—C77	1.509 (8)
C22—H22A	0.9700	C76—H76A	0.9700
C22—H22B	0.9700	C76—H76B	0.9700
C23—C24	1.507 (8)	C77—C78	1.526 (8)
C23—H23A	0.9700	C77—H77A	0.9700
C23—H23B	0.9700	C77—H77B	0.9700
C24—H24A	0.9700	C78—H78A	0.9700
C24—H24B	0.9700	C78—H78B	0.9700
C25—C30	1.528 (7)	C79—C80	1.397 (8)
C25—C26	1.528 (7)	C79—C84	1.397 (8)
C25—H25	0.9800	C80—C81	1.392 (8)
C26—C27	1.520 (7)	C80—H80	0.9300
C26—H26A	0.9700	C81—C82	1.366 (9)
C26—H26B	0.9700	C81—H81	0.9300
C27—C28	1.523 (9)	C82—C83	1.391 (9)
C27—H27A	0.9700	C82—H82	0.9300
C27—H27B	0.9700	C83—C84	1.364 (8)
C28—C29	1.503 (10)	C83—H83	0.9300
C28—H28A	0.9700	C85—C86	1.528 (8)
C28—H28B	0.9700	C85—C90	1.530 (8)
C29—C30	1.528 (7)	C85—H85	0.9800
C29—H29A	0.9700	C86—C87	1.541 (8)
C29—H29B	0.9700	C86—H86A	0.9700
C30—H30A	0.9700	C86—H86B	0.9700
C30—H30B	0.9700	C87—C88	1.532 (9)
C31—C36	1.537 (7)	C87—H87A	0.9700
C31—C32	1.540 (7)	C87—H87B	0.9700
C31—H31	0.9800	C88—C89	1.499 (9)
C32—C33	1.536 (8)	C88—H88A	0.9700
C32—H32A	0.9700	C88—H88B	0.9700
C32—H32B	0.9700	C89—C90	1.530 (8)
C33—C34	1.518 (9)	C89—H89A	0.9700
C33—H33A	0.9700	C89—H89B	0.9700
C33—H33B	0.9700	C90—H90A	0.9700
C34—C35	1.505 (9)	C90—H90B	0.9700
C34—H34A	0.9700	C91—H91A	0.9600
C34—H34B	0.9700	C91—H91B	0.9600
C35—C36	1.518 (7)	C91—H91C	0.9600
C35—H35A	0.9700	C92—H92A	0.9600
C35—H35B	0.9700	C92—H92B	0.9600
C36—H36A	0.9700	C92—H92C	0.9600
C36—H36B	0.9700	C93—H93A	0.9600
C37—C38	1.529 (7)	C93—H93B	0.9600
C37—C42	1.532 (6)	C93—H93C	0.9600
C37—H37	0.9800	C94—H94A	0.9600
C38—C39	1.521 (7)	C94—H94B	0.9600
C38—H38A	0.9700	C94—H94C	0.9600
			
P2—Ru1—P1	173.08 (5)	C40—C39—H39B	109.2
P2—Ru1—Cl1	90.86 (5)	C38—C39—H39B	109.2
P1—Ru1—Cl1	93.82 (5)	H39A—C39—H39B	107.9
P2—Ru1—Cl2	94.63 (5)	C39—C40—C41	110.6 (4)
P1—Ru1—Cl2	90.67 (5)	C39—C40—H40A	109.5
Cl1—Ru1—Cl2	87.48 (5)	C41—C40—H40A	109.5
P2—Ru1—Br1	82.87 (4)	C39—C40—H40B	109.5
P1—Ru1—Br1	91.73 (4)	C41—C40—H40B	109.5
Cl1—Ru1—Br1	93.73 (4)	H40A—C40—H40B	108.1
Cl2—Ru1—Br1	177.22 (4)	C40—C41—C42	111.7 (4)
P2—Ru1—Br2	92.70 (4)	C40—C41—H41A	109.3
P1—Ru1—Br2	82.54 (4)	C42—C41—H41A	109.3
Cl1—Ru1—Br2	176.29 (4)	C40—C41—H41B	109.3
Cl2—Ru1—Br2	93.26 (4)	C42—C41—H41B	109.3
Br1—Ru1—Br2	85.69 (2)	H41A—C41—H41B	107.9
P3—Ru2—P4	173.11 (5)	C41—C42—C37	110.6 (4)
P3—Ru2—Cl3	93.94 (5)	C41—C42—H42A	109.5
P4—Ru2—Cl3	90.46 (5)	C37—C42—H42A	109.5
P3—Ru2—Cl4	89.60 (5)	C41—C42—H42B	109.5
P4—Ru2—Cl4	95.86 (5)	C37—C42—H42B	109.5
Cl3—Ru2—Cl4	88.10 (5)	H42A—C42—H42B	108.1
P3—Ru2—Br3	92.06 (4)	C48—C43—C44	116.0 (6)
P4—Ru2—Br3	82.41 (4)	C48—C43—P4	119.3 (4)
Cl3—Ru2—Br3	92.90 (4)	C44—C43—P4	124.2 (5)
Cl4—Ru2—Br3	178.00 (4)	C45—C44—C43	120.9 (6)
P3—Ru2—Br4	82.78 (4)	C45—C44—H44	119.5
P4—Ru2—Br4	92.73 (4)	C43—C44—H44	119.5
Cl3—Ru2—Br4	176.60 (4)	C46—C45—C44	121.2 (6)
Cl4—Ru2—Br4	92.73 (4)	C46—C45—H45	119.4
Br3—Ru2—Br4	86.38 (2)	C44—C45—H45	119.4
Cl5^i^—Ru3—Cl5	87.30 (9)	C45—C46—C47	119.8 (7)
Cl5^i^—Ru3—P5^i^	97.09 (6)	C45—C46—H46	120.1
Cl5—Ru3—P5^i^	87.64 (6)	C47—C46—H46	120.1
Cl5^i^—Ru3—P5	87.64 (6)	C46—C47—C48	118.3 (7)
Cl5—Ru3—P5	97.09 (6)	C46—C47—H47	120.8
P5^i^—Ru3—P5	173.48 (8)	C48—C47—H47	120.8
Cl5^i^—Ru3—Br5	94.62 (5)	C47—C48—C43	123.6 (6)
Cl5—Ru3—Br5	176.85 (5)	C47—C48—Br3	117.5 (5)
P5^i^—Ru3—Br5	94.60 (4)	C43—C48—Br3	118.9 (4)
P5—Ru3—Br5	80.51 (4)	C54—C49—C50	116.2 (5)
Cl5^i^—Ru3—Br5^i^	176.85 (5)	C54—C49—P3	119.6 (4)
Cl5—Ru3—Br5^i^	94.62 (5)	C50—C49—P3	123.9 (4)
P5^i^—Ru3—Br5^i^	80.51 (4)	C51—C50—C49	121.8 (5)
P5—Ru3—Br5^i^	94.59 (4)	C51—C50—H50	119.1
Br5—Ru3—Br5^i^	83.58 (4)	C49—C50—H50	119.1
C12—Br1—Ru1	101.88 (16)	C50—C51—C52	119.6 (6)
C18—Br2—Ru1	100.45 (16)	C50—C51—H51	120.2
C48—Br3—Ru2	101.92 (18)	C52—C51—H51	120.2
C54—Br4—Ru2	100.96 (16)	C51—C52—C53	120.6 (6)
C84—Br5—Ru3	99.74 (17)	C51—C52—H52	119.7
C13—P1—C19	102.8 (2)	C53—C52—H52	119.7
C13—P1—C1	101.7 (2)	C54—C53—C52	117.7 (5)
C19—P1—C1	103.9 (2)	C54—C53—H53	121.1
C13—P1—Ru1	104.95 (17)	C52—C53—H53	121.1
C19—P1—Ru1	120.52 (19)	C53—C54—C49	124.0 (5)
C1—P1—Ru1	120.05 (17)	C53—C54—Br4	117.6 (4)
C7—P2—C25	105.3 (2)	C49—C54—Br4	118.4 (4)
C7—P2—C31	100.6 (2)	C60—C55—C56	110.2 (4)
C25—P2—C31	103.5 (2)	C60—C55—P3	111.9 (3)
C7—P2—Ru1	105.69 (18)	C56—C55—P3	110.8 (3)
C25—P2—Ru1	119.25 (18)	C60—C55—H55	108.0
C31—P2—Ru1	120.09 (17)	C56—C55—H55	108.0
C49—P3—C55	105.0 (2)	P3—C55—H55	108.0
C49—P3—C37	100.0 (2)	C57—C56—C55	110.9 (5)
C55—P3—C37	103.0 (2)	C57—C56—H56A	109.5
C49—P3—Ru2	105.41 (17)	C55—C56—H56A	109.5
C55—P3—Ru2	121.00 (17)	C57—C56—H56B	109.5
C37—P3—Ru2	119.63 (16)	C55—C56—H56B	109.5
C43—P4—C61	105.3 (3)	H56A—C56—H56B	108.0
C43—P4—C67	99.8 (2)	C58—C57—C56	111.4 (5)
C61—P4—C67	104.1 (2)	C58—C57—H57A	109.3
C43—P4—Ru2	106.10 (18)	C56—C57—H57A	109.3
C61—P4—Ru2	120.37 (19)	C58—C57—H57B	109.3
C67—P4—Ru2	118.58 (17)	C56—C57—H57B	109.3
C73—P5—C79	104.9 (3)	H57A—C57—H57B	108.0
C73—P5—C85	103.4 (3)	C59—C58—C57	110.8 (5)
C79—P5—C85	101.8 (3)	C59—C58—H58A	109.5
C73—P5—Ru3	118.7 (2)	C57—C58—H58A	109.5
C79—P5—Ru3	105.30 (19)	C59—C58—H58B	109.5
C85—P5—Ru3	120.47 (19)	C57—C58—H58B	109.5
C91—O1—H1	109.5	H58A—C58—H58B	108.1
C92—O2—H2	109.5	C58—C59—C60	112.4 (5)
C93—O3—H3	109.5	C58—C59—H59A	109.1
C94—O4—H4	109.5	C60—C59—H59A	109.1
C6—C1—C2	109.1 (4)	C58—C59—H59B	109.1
C6—C1—P1	111.6 (4)	C60—C59—H59B	109.1
C2—C1—P1	115.0 (4)	H59A—C59—H59B	107.8
C6—C1—H1A	106.9	C55—C60—C59	110.3 (5)
C2—C1—H1A	106.9	C55—C60—H60A	109.6
P1—C1—H1A	106.9	C59—C60—H60A	109.6
C3—C2—C1	111.5 (5)	C55—C60—H60B	109.6
C3—C2—H2A	109.3	C59—C60—H60B	109.6
C1—C2—H2A	109.3	H60A—C60—H60B	108.1
C3—C2—H2B	109.3	C62—C61—C66	110.4 (5)
C1—C2—H2B	109.3	C62—C61—P4	110.5 (4)
H2A—C2—H2B	108.0	C66—C61—P4	112.5 (4)
C4—C3—C2	112.0 (5)	C62—C61—H61	107.7
C4—C3—H3A	109.2	C66—C61—H61	107.7
C2—C3—H3A	109.2	P4—C61—H61	107.7
C4—C3—H3B	109.2	C61—C62—C63	111.7 (5)
C2—C3—H3B	109.2	C61—C62—H62A	109.3
H3A—C3—H3B	107.9	C63—C62—H62A	109.3
C3—C4—C5	110.5 (5)	C61—C62—H62B	109.3
C3—C4—H4A	109.6	C63—C62—H62B	109.3
C5—C4—H4A	109.6	H62A—C62—H62B	107.9
C3—C4—H4B	109.6	C64—C63—C62	110.3 (5)
C5—C4—H4B	109.6	C64—C63—H63A	109.6
H4A—C4—H4B	108.1	C62—C63—H63A	109.6
C4—C5—C6	112.1 (5)	C64—C63—H63B	109.6
C4—C5—H5A	109.2	C62—C63—H63B	109.6
C6—C5—H5A	109.2	H63A—C63—H63B	108.1
C4—C5—H5B	109.2	C63—C64—C65	111.1 (6)
C6—C5—H5B	109.2	C63—C64—H64A	109.4
H5A—C5—H5B	107.9	C65—C64—H64A	109.4
C5—C6—C1	111.2 (5)	C63—C64—H64B	109.4
C5—C6—H6A	109.4	C65—C64—H64B	109.4
C1—C6—H6A	109.4	H64A—C64—H64B	108.0
C5—C6—H6B	109.4	C64—C65—C66	111.5 (6)
C1—C6—H6B	109.4	C64—C65—H65A	109.3
H6A—C6—H6B	108.0	C66—C65—H65A	109.3
C12—C7—C8	115.8 (5)	C64—C65—H65B	109.3
C12—C7—P2	119.8 (4)	C66—C65—H65B	109.3
C8—C7—P2	123.8 (4)	H65A—C65—H65B	108.0
C9—C8—C7	120.8 (6)	C65—C66—C61	111.4 (5)
C9—C8—H8	119.6	C65—C66—H66A	109.3
C7—C8—H8	119.6	C61—C66—H66A	109.3
C10—C9—C8	120.6 (6)	C65—C66—H66B	109.3
C10—C9—H9	119.7	C61—C66—H66B	109.3
C8—C9—H9	119.7	H66A—C66—H66B	108.0
C9—C10—C11	120.2 (6)	C72—C67—C68	108.7 (4)
C9—C10—H10	119.9	C72—C67—P4	116.0 (4)
C11—C10—H10	119.9	C68—C67—P4	113.0 (4)
C12—C11—C10	118.0 (6)	C72—C67—H67	106.1
C12—C11—H11	121.0	C68—C67—H67	106.1
C10—C11—H11	121.0	P4—C67—H67	106.1
C11—C12—C7	124.5 (5)	C69—C68—C67	111.6 (5)
C11—C12—Br1	116.7 (4)	C69—C68—H68A	109.3
C7—C12—Br1	118.8 (4)	C67—C68—H68A	109.3
C18—C13—C14	115.9 (5)	C69—C68—H68B	109.3
C18—C13—P1	119.6 (4)	C67—C68—H68B	109.3
C14—C13—P1	124.5 (4)	H68A—C68—H68B	108.0
C15—C14—C13	121.2 (5)	C68—C69—C70	110.8 (5)
C15—C14—H14	119.4	C68—C69—H69A	109.5
C13—C14—H14	119.4	C70—C69—H69A	109.5
C16—C15—C14	120.1 (6)	C68—C69—H69B	109.5
C16—C15—H15	120.0	C70—C69—H69B	109.5
C14—C15—H15	120.0	H69A—C69—H69B	108.1
C15—C16—C17	120.9 (6)	C69—C70—C71	111.7 (5)
C15—C16—H16	119.6	C69—C70—H70A	109.3
C17—C16—H16	119.6	C71—C70—H70A	109.3
C16—C17—C18	117.8 (6)	C69—C70—H70B	109.3
C16—C17—H17	121.1	C71—C70—H70B	109.3
C18—C17—H17	121.1	H70A—C70—H70B	107.9
C17—C18—C13	124.2 (5)	C72—C71—C70	111.2 (5)
C17—C18—Br2	117.1 (4)	C72—C71—H71A	109.4
C13—C18—Br2	118.6 (4)	C70—C71—H71A	109.4
C24—C19—C20	110.4 (5)	C72—C71—H71B	109.4
C24—C19—P1	112.1 (4)	C70—C71—H71B	109.4
C20—C19—P1	111.6 (4)	H71A—C71—H71B	108.0
C24—C19—H19	107.5	C71—C72—C67	111.0 (5)
C20—C19—H19	107.5	C71—C72—H72A	109.4
P1—C19—H19	107.5	C67—C72—H72A	109.4
C21—C20—C19	108.9 (6)	C71—C72—H72B	109.4
C21—C20—H20A	109.9	C67—C72—H72B	109.4
C19—C20—H20A	109.9	H72A—C72—H72B	108.0
C21—C20—H20B	109.9	C74—C73—C78	111.1 (5)
C19—C20—H20B	109.9	C74—C73—P5	110.8 (4)
H20A—C20—H20B	108.3	C78—C73—P5	109.3 (4)
C22—C21—C20	112.0 (6)	C74—C73—H73	108.5
C22—C21—H21A	109.2	C78—C73—H73	108.5
C20—C21—H21A	109.2	P5—C73—H73	108.5
C22—C21—H21B	109.2	C75—C74—C73	111.2 (5)
C20—C21—H21B	109.2	C75—C74—H74A	109.4
H21A—C21—H21B	107.9	C73—C74—H74A	109.4
C21—C22—C23	112.4 (7)	C75—C74—H74B	109.4
C21—C22—H22A	109.1	C73—C74—H74B	109.4
C23—C22—H22A	109.1	H74A—C74—H74B	108.0
C21—C22—H22B	109.1	C74—C75—C76	110.9 (5)
C23—C22—H22B	109.1	C74—C75—H75A	109.5
H22A—C22—H22B	107.9	C76—C75—H75A	109.5
C24—C23—C22	110.8 (6)	C74—C75—H75B	109.5
C24—C23—H23A	109.5	C76—C75—H75B	109.5
C22—C23—H23A	109.5	H75A—C75—H75B	108.0
C24—C23—H23B	109.5	C77—C76—C75	110.1 (5)
C22—C23—H23B	109.5	C77—C76—H76A	109.6
H23A—C23—H23B	108.1	C75—C76—H76A	109.6
C23—C24—C19	110.5 (5)	C77—C76—H76B	109.6
C23—C24—H24A	109.6	C75—C76—H76B	109.6
C19—C24—H24A	109.6	H76A—C76—H76B	108.1
C23—C24—H24B	109.6	C76—C77—C78	112.1 (5)
C19—C24—H24B	109.6	C76—C77—H77A	109.2
H24A—C24—H24B	108.1	C78—C77—H77A	109.2
C30—C25—C26	111.0 (4)	C76—C77—H77B	109.2
C30—C25—P2	112.1 (4)	C78—C77—H77B	109.2
C26—C25—P2	111.1 (4)	H77A—C77—H77B	107.9
C30—C25—H25	107.5	C77—C78—C73	111.9 (5)
C26—C25—H25	107.5	C77—C78—H78A	109.2
P2—C25—H25	107.5	C73—C78—H78A	109.2
C27—C26—C25	110.4 (5)	C77—C78—H78B	109.2
C27—C26—H26A	109.6	C73—C78—H78B	109.2
C25—C26—H26A	109.6	H78A—C78—H78B	107.9
C27—C26—H26B	109.6	C80—C79—C84	116.6 (5)
C25—C26—H26B	109.6	C80—C79—P5	125.1 (5)
H26A—C26—H26B	108.1	C84—C79—P5	118.3 (4)
C26—C27—C28	110.7 (5)	C81—C80—C79	121.2 (6)
C26—C27—H27A	109.5	C81—C80—H80	119.4
C28—C27—H27A	109.5	C79—C80—H80	119.4
C26—C27—H27B	109.5	C82—C81—C80	120.1 (6)
C28—C27—H27B	109.5	C82—C81—H81	120.0
H27A—C27—H27B	108.1	C80—C81—H81	120.0
C29—C28—C27	111.4 (6)	C81—C82—C83	120.0 (6)
C29—C28—H28A	109.3	C81—C82—H82	120.0
C27—C28—H28A	109.3	C83—C82—H82	120.0
C29—C28—H28B	109.3	C84—C83—C82	119.5 (6)
C27—C28—H28B	109.3	C84—C83—H83	120.3
H28A—C28—H28B	108.0	C82—C83—H83	120.3
C28—C29—C30	111.0 (6)	C83—C84—C79	122.6 (6)
C28—C29—H29A	109.4	C83—C84—Br5	119.1 (5)
C30—C29—H29A	109.4	C79—C84—Br5	118.3 (4)
C28—C29—H29B	109.4	C86—C85—C90	110.0 (5)
C30—C29—H29B	109.4	C86—C85—P5	114.3 (4)
H29A—C29—H29B	108.0	C90—C85—P5	112.7 (4)
C29—C30—C25	111.2 (5)	C86—C85—H85	106.4
C29—C30—H30A	109.4	C90—C85—H85	106.4
C25—C30—H30A	109.4	P5—C85—H85	106.4
C29—C30—H30B	109.4	C85—C86—C87	110.2 (5)
C25—C30—H30B	109.4	C85—C86—H86A	109.6
H30A—C30—H30B	108.0	C87—C86—H86A	109.6
C36—C31—C32	108.0 (4)	C85—C86—H86B	109.6
C36—C31—P2	112.1 (4)	C87—C86—H86B	109.6
C32—C31—P2	116.4 (4)	H86A—C86—H86B	108.1
C36—C31—H31	106.6	C88—C87—C86	111.5 (6)
C32—C31—H31	106.6	C88—C87—H87A	109.3
P2—C31—H31	106.6	C86—C87—H87A	109.3
C33—C32—C31	111.4 (5)	C88—C87—H87B	109.3
C33—C32—H32A	109.4	C86—C87—H87B	109.3
C31—C32—H32A	109.4	H87A—C87—H87B	108.0
C33—C32—H32B	109.4	C89—C88—C87	109.8 (6)
C31—C32—H32B	109.4	C89—C88—H88A	109.7
H32A—C32—H32B	108.0	C87—C88—H88A	109.7
C34—C33—C32	110.5 (5)	C89—C88—H88B	109.7
C34—C33—H33A	109.6	C87—C88—H88B	109.7
C32—C33—H33A	109.6	H88A—C88—H88B	108.2
C34—C33—H33B	109.6	C88—C89—C90	112.2 (6)
C32—C33—H33B	109.6	C88—C89—H89A	109.2
H33A—C33—H33B	108.1	C90—C89—H89A	109.2
C35—C34—C33	111.2 (6)	C88—C89—H89B	109.2
C35—C34—H34A	109.4	C90—C89—H89B	109.2
C33—C34—H34A	109.4	H89A—C89—H89B	107.9
C35—C34—H34B	109.4	C89—C90—C85	111.2 (5)
C33—C34—H34B	109.4	C89—C90—H90A	109.4
H34A—C34—H34B	108.0	C85—C90—H90A	109.4
C34—C35—C36	111.0 (5)	C89—C90—H90B	109.4
C34—C35—H35A	109.4	C85—C90—H90B	109.4
C36—C35—H35A	109.4	H90A—C90—H90B	108.0
C34—C35—H35B	109.4	O1—C91—H91A	109.5
C36—C35—H35B	109.4	O1—C91—H91B	109.5
H35A—C35—H35B	108.0	H91A—C91—H91B	109.5
C35—C36—C31	111.2 (5)	O1—C91—H91C	109.5
C35—C36—H36A	109.4	H91A—C91—H91C	109.5
C31—C36—H36A	109.4	H91B—C91—H91C	109.5
C35—C36—H36B	109.4	O2—C92—H92A	109.5
C31—C36—H36B	109.4	O2—C92—H92B	109.5
H36A—C36—H36B	108.0	H92A—C92—H92B	109.5
C38—C37—C42	108.2 (4)	O2—C92—H92C	109.5
C38—C37—P3	114.6 (3)	H92A—C92—H92C	109.5
C42—C37—P3	113.0 (3)	H92B—C92—H92C	109.5
C38—C37—H37	106.9	O3—C93—H93A	109.5
C42—C37—H37	106.9	O3—C93—H93B	109.5
P3—C37—H37	106.9	H93A—C93—H93B	109.5
C39—C38—C37	111.7 (4)	O3—C93—H93C	109.5
C39—C38—H38A	109.3	H93A—C93—H93C	109.5
C37—C38—H38A	109.3	H93B—C93—H93C	109.5
C39—C38—H38B	109.3	O4—C94—H94A	109.5
C37—C38—H38B	109.3	O4—C94—H94B	109.5
H38A—C38—H38B	107.9	H94A—C94—H94B	109.5
C40—C39—C38	112.0 (5)	O4—C94—H94C	109.5
C40—C39—H39A	109.2	H94A—C94—H94C	109.5
C38—C39—H39A	109.2	H94B—C94—H94C	109.5
			
C13—P1—C1—C6	55.5 (4)	C44—C45—C46—C47	−3.4 (11)
C19—P1—C1—C6	162.0 (4)	C45—C46—C47—C48	2.2 (10)
Ru1—P1—C1—C6	−59.6 (4)	C46—C47—C48—C43	0.9 (9)
C13—P1—C1—C2	−69.5 (5)	C46—C47—C48—Br3	179.2 (5)
C19—P1—C1—C2	37.0 (5)	C44—C43—C48—C47	−2.7 (8)
Ru1—P1—C1—C2	175.4 (3)	P4—C43—C48—C47	170.1 (5)
C6—C1—C2—C3	56.2 (7)	C44—C43—C48—Br3	179.0 (4)
P1—C1—C2—C3	−177.5 (4)	P4—C43—C48—Br3	−8.1 (6)
C1—C2—C3—C4	−56.5 (7)	C55—P3—C49—C54	161.3 (4)
C2—C3—C4—C5	54.8 (8)	C37—P3—C49—C54	−92.3 (4)
C3—C4—C5—C6	−54.9 (7)	Ru2—P3—C49—C54	32.4 (5)
C4—C5—C6—C1	56.7 (7)	C55—P3—C49—C50	−24.0 (5)
C2—C1—C6—C5	−56.1 (6)	C37—P3—C49—C50	82.4 (5)
P1—C1—C6—C5	175.7 (4)	Ru2—P3—C49—C50	−152.8 (4)
C25—P2—C7—C12	156.2 (4)	C54—C49—C50—C51	1.9 (8)
C31—P2—C7—C12	−96.5 (5)	P3—C49—C50—C51	−173.0 (4)
Ru1—P2—C7—C12	29.1 (5)	C49—C50—C51—C52	0.8 (9)
C25—P2—C7—C8	−32.7 (5)	C50—C51—C52—C53	−2.0 (9)
C31—P2—C7—C8	74.6 (5)	C51—C52—C53—C54	0.4 (9)
Ru1—P2—C7—C8	−159.7 (4)	C52—C53—C54—C49	2.5 (9)
C12—C7—C8—C9	−1.0 (8)	C52—C53—C54—Br4	−176.8 (4)
P2—C7—C8—C9	−172.5 (4)	C50—C49—C54—C53	−3.6 (8)
C7—C8—C9—C10	0.9 (9)	P3—C49—C54—C53	171.5 (4)
C8—C9—C10—C11	−0.5 (9)	C50—C49—C54—Br4	175.7 (4)
C9—C10—C11—C12	0.3 (9)	P3—C49—C54—Br4	−9.1 (6)
C10—C11—C12—C7	−0.5 (9)	C49—P3—C55—C60	−72.8 (4)
C10—C11—C12—Br1	179.6 (4)	C37—P3—C55—C60	−177.1 (4)
C8—C7—C12—C11	0.8 (8)	Ru2—P3—C55—C60	46.0 (4)
P2—C7—C12—C11	172.7 (4)	C49—P3—C55—C56	163.9 (4)
C8—C7—C12—Br1	−179.2 (4)	C37—P3—C55—C56	59.6 (4)
P2—C7—C12—Br1	−7.4 (6)	Ru2—P3—C55—C56	−77.4 (4)
C19—P1—C13—C18	156.0 (4)	C60—C55—C56—C57	56.3 (6)
C1—P1—C13—C18	−96.6 (4)	P3—C55—C56—C57	−179.4 (4)
Ru1—P1—C13—C18	29.2 (4)	C55—C56—C57—C58	−57.1 (7)
C19—P1—C13—C14	−23.3 (5)	C56—C57—C58—C59	56.7 (7)
C1—P1—C13—C14	84.1 (5)	C57—C58—C59—C60	−55.9 (7)
Ru1—P1—C13—C14	−150.1 (4)	C56—C55—C60—C59	−54.7 (6)
C18—C13—C14—C15	0.4 (8)	P3—C55—C60—C59	−178.4 (4)
P1—C13—C14—C15	179.7 (4)	C58—C59—C60—C55	55.6 (7)
C13—C14—C15—C16	1.0 (9)	C43—P4—C61—C62	164.2 (4)
C14—C15—C16—C17	−1.7 (10)	C67—P4—C61—C62	59.7 (4)
C15—C16—C17—C18	0.8 (9)	Ru2—P4—C61—C62	−76.2 (4)
C16—C17—C18—C13	0.8 (9)	C43—P4—C61—C66	−71.9 (5)
C16—C17—C18—Br2	−176.8 (5)	C67—P4—C61—C66	−176.4 (4)
C14—C13—C18—C17	−1.3 (8)	Ru2—P4—C61—C66	47.7 (5)
P1—C13—C18—C17	179.3 (4)	C66—C61—C62—C63	55.0 (6)
C14—C13—C18—Br2	176.2 (4)	P4—C61—C62—C63	−179.9 (4)
P1—C13—C18—Br2	−3.2 (6)	C61—C62—C63—C64	−56.7 (7)
C13—P1—C19—C24	−69.3 (4)	C62—C63—C64—C65	56.9 (7)
C1—P1—C19—C24	−175.0 (4)	C63—C64—C65—C66	−57.0 (7)
Ru1—P1—C19—C24	46.9 (5)	C64—C65—C66—C61	55.4 (7)
C13—P1—C19—C20	166.2 (4)	C62—C61—C66—C65	−54.3 (7)
C1—P1—C19—C20	60.6 (5)	P4—C61—C66—C65	−178.2 (5)
Ru1—P1—C19—C20	−77.6 (4)	C43—P4—C67—C72	−72.2 (5)
C24—C19—C20—C21	57.9 (7)	C61—P4—C67—C72	36.4 (5)
P1—C19—C20—C21	−176.8 (5)	Ru2—P4—C67—C72	173.3 (3)
C19—C20—C21—C22	−55.6 (8)	C43—P4—C67—C68	54.3 (4)
C20—C21—C22—C23	54.6 (9)	C61—P4—C67—C68	162.8 (4)
C21—C22—C23—C24	−54.5 (9)	Ru2—P4—C67—C68	−60.2 (4)
C22—C23—C24—C19	56.6 (8)	C72—C67—C68—C69	−58.7 (6)
C20—C19—C24—C23	−59.4 (7)	P4—C67—C68—C69	171.0 (4)
P1—C19—C24—C23	175.5 (5)	C67—C68—C69—C70	57.2 (7)
C7—P2—C25—C30	−73.3 (4)	C68—C69—C70—C71	−54.3 (8)
C31—P2—C25—C30	−178.5 (4)	C69—C70—C71—C72	54.1 (8)
Ru1—P2—C25—C30	45.0 (4)	C70—C71—C72—C67	−56.4 (7)
C7—P2—C25—C26	161.8 (4)	C68—C67—C72—C71	57.9 (6)
C31—P2—C25—C26	56.6 (4)	P4—C67—C72—C71	−173.4 (4)
Ru1—P2—C25—C26	−79.9 (4)	C79—P5—C73—C74	70.4 (4)
C30—C25—C26—C27	56.1 (6)	C85—P5—C73—C74	176.7 (4)
P2—C25—C26—C27	−178.5 (4)	Ru3—P5—C73—C74	−46.8 (4)
C25—C26—C27—C28	−57.0 (8)	C79—P5—C73—C78	−166.9 (4)
C26—C27—C28—C29	57.5 (8)	C85—P5—C73—C78	−60.6 (5)
C27—C28—C29—C30	−56.2 (8)	Ru3—P5—C73—C78	75.9 (4)
C28—C29—C30—C25	55.0 (7)	C78—C73—C74—C75	53.7 (7)
C26—C25—C30—C29	−55.0 (6)	P5—C73—C74—C75	175.3 (4)
P2—C25—C30—C29	−179.9 (4)	C73—C74—C75—C76	−57.9 (7)
C7—P2—C31—C36	56.6 (4)	C74—C75—C76—C77	59.1 (7)
C25—P2—C31—C36	165.3 (4)	C75—C76—C77—C78	−56.8 (7)
Ru1—P2—C31—C36	−58.6 (4)	C76—C77—C78—C73	53.5 (7)
C7—P2—C31—C32	−68.4 (4)	C74—C73—C78—C77	−51.2 (7)
C25—P2—C31—C32	40.3 (5)	P5—C73—C78—C77	−173.8 (4)
Ru1—P2—C31—C32	176.4 (3)	C73—P5—C79—C80	22.0 (6)
C36—C31—C32—C33	57.5 (6)	C85—P5—C79—C80	−85.6 (5)
P2—C31—C32—C33	−175.3 (4)	Ru3—P5—C79—C80	148.0 (5)
C31—C32—C33—C34	−57.0 (8)	C73—P5—C79—C84	−155.7 (5)
C32—C33—C34—C35	55.6 (8)	C85—P5—C79—C84	96.7 (5)
C33—C34—C35—C36	−56.7 (8)	Ru3—P5—C79—C84	−29.7 (5)
C34—C35—C36—C31	58.7 (7)	C84—C79—C80—C81	2.4 (9)
C32—C31—C36—C35	−58.2 (6)	P5—C79—C80—C81	−175.4 (5)
P2—C31—C36—C35	172.2 (4)	C79—C80—C81—C82	−1.5 (10)
C49—P3—C37—C38	−68.7 (4)	C80—C81—C82—C83	−0.1 (10)
C55—P3—C37—C38	39.4 (4)	C81—C82—C83—C84	0.6 (10)
Ru2—P3—C37—C38	177.0 (3)	C82—C83—C84—C79	0.4 (10)
C49—P3—C37—C42	55.9 (4)	C82—C83—C84—Br5	177.7 (5)
C55—P3—C37—C42	163.9 (4)	C80—C79—C84—C83	−1.9 (9)
Ru2—P3—C37—C42	−58.4 (4)	P5—C79—C84—C83	176.1 (5)
C42—C37—C38—C39	58.4 (6)	C80—C79—C84—Br5	−179.2 (4)
P3—C37—C38—C39	−174.6 (4)	P5—C79—C84—Br5	−1.3 (6)
C37—C38—C39—C40	−56.4 (6)	C73—P5—C85—C86	−42.5 (5)
C38—C39—C40—C41	53.0 (7)	C79—P5—C85—C86	66.1 (5)
C39—C40—C41—C42	−54.2 (7)	Ru3—P5—C85—C86	−178.1 (4)
C40—C41—C42—C37	58.2 (6)	C73—P5—C85—C90	−169.1 (4)
C38—C37—C42—C41	−59.0 (6)	C79—P5—C85—C90	−60.4 (5)
P3—C37—C42—C41	173.0 (4)	Ru3—P5—C85—C90	55.4 (5)
C61—P4—C43—C48	158.7 (4)	C90—C85—C86—C87	−56.4 (7)
C67—P4—C43—C48	−93.6 (5)	P5—C85—C86—C87	175.7 (5)
Ru2—P4—C43—C48	30.1 (5)	C85—C86—C87—C88	57.5 (8)
C61—P4—C43—C44	−29.0 (5)	C86—C87—C88—C89	−56.7 (9)
C67—P4—C43—C44	78.7 (5)	C87—C88—C89—C90	56.0 (8)
Ru2—P4—C43—C44	−157.6 (4)	C88—C89—C90—C85	−56.7 (8)
C48—C43—C44—C45	1.6 (9)	C86—C85—C90—C89	56.0 (7)
P4—C43—C44—C45	−170.9 (5)	P5—C85—C90—C89	−175.2 (4)
C43—C44—C45—C46	1.4 (10)		

Symmetry code: (i) −*x*+1/2, *y*, −*z*+3/2.

### Bis[(2-bromophenyl)dicyclohexylphosphane-κ2Br,P]dichloridoruthenium(II) methanol 1.6-solvate Hydrogen-bond geometry (Å, º)

**Table:**

*D*—H···*A*	*D*—H	H···*A*	*D*···*A*	*D*—H···*A*
O1—H1···Cl2	0.82	2.47	3.243 (6)	157
O2—H2···O1	0.82	2.29	2.731 (10)	114
O3—H3···Cl1	0.82	2.44	3.259 (7)	172
O4—H4···O2	0.82	2.42	2.735 (12)	104
C6—H6*B*···Cl2	0.97	2.70	3.453 (6)	135
C11—H11···Cl5^i^	0.93	2.73	3.474 (6)	138
C20—H20*B*···Cl1	0.97	2.67	3.511 (6)	146
C24—H24*A*···Br1	0.97	2.89	3.378 (6)	112
C26—H26*B*···Cl2	0.97	2.69	3.532 (6)	146
C30—H30*A*···Br2	0.97	2.82	3.359 (5)	116
C36—H36*B*···Cl1	0.97	2.65	3.406 (6)	135
C42—H42*B*···Cl4	0.97	2.66	3.388 (6)	133
C56—H56*B*···Cl3	0.97	2.64	3.495 (6)	147
C60—H60*B*···Br3	0.97	2.89	3.429 (6)	116
C62—H62*A*···Cl4	0.97	2.68	3.547 (6)	150
C66—H66*A*···Br4	0.97	2.86	3.446 (6)	120
C68—H68*B*···Cl3	0.97	2.70	3.414 (6)	131
C78—H78*A*···Cl5	0.97	2.67	3.531 (6)	148
C90—H90*A*···Cl5^i^	0.97	2.65	3.395 (7)	134

Symmetry code: (i) −*x*+1/2, *y*, −*z*+3/2.
